# Exploring the Relationship between Co-Sleeping, Maternal Mental Health and Expression of Complaints during Infancy, and Breastfeeding

**DOI:** 10.3390/healthcare12131278

**Published:** 2024-06-26

**Authors:** Marilena Vogiatzoglou, Maria Iliadou, Evangelia Antoniou, Ewa Andersson, Irina Mrvoljak-Theodoropoulou, Calliope Dagla, Dimitra Sotiropoulou, Eleni Tsolaridou, Eirini Orovou, Eirini Tomara, Maria Dagla

**Affiliations:** 1 Department of Midwifery, School of Health and Care Sciences, University of West Attica, 12243 Athens, Greece; m.vogiatzoglou@fainareti.gr (M.V.); miliad@uniwa.gr (M.I.); lilanton@uniwa.gr (E.A.); etsolaridou@uniwa.gr (E.T.); eorovou@uniwa.gr (E.O.); etomara@uniwa.gr (E.T.); 2 Day Center for the Care of the Mental Health of Women (Perinatal Mental Health Disorders), Non-Profit Organization “FAINARETI”, 17121 Athens, Greece; imrvoljak@hotmail.com (I.M.-T.); k.dagla@fainareti.gr (C.D.); d.sotiropoulou@fainareti.gr (D.S.); 3 Department of Women’s and Children’s Health, Karolinska Institutet, 17177 Stockholm, Sweden; ewa.andersson@ki.se; 4 Department of Midwifery, University of Western Macedonia, 50200 Ptolemaida, Greece

**Keywords:** breastfeeding, co-sleeping, maternal complaints, sleep, infancy, mothers’ difficulties, maternal mental health, sleep quality

## Abstract

Background: This study explores whether the implementation of co-sleeping in infants aged 6–12 months (a) is associated with maternal complaints and mothers’ difficulties regarding their infant’s sleep, (b) is associated with maternal mental health, (c) affects infant sleep characteristics and maternal sleep quality, and (d) is associated with breastfeeding. Methods: This study is a cross-sectional study conducted from July to November 2021. A total of 151 new mothers of infants aged 6–12 months participated. All participants were divided into two different groups, the group of mothers who adopted the co-sleeping method from birth up to the time of the survey and the group of those who did not adopt co-sleeping at that time. The Brief Infant Sleep Questionnaire—Revised Short Form (BISQ-R SF), the Edinburgh Postnatal Depression Scale (EPDS), the State-Trait Anxiety Inventory (STAI), and a questionnaire on mothers’ demographics were administered. Results: Regarding the mothers’ complaints, mothers who co-sleep with their children have lower sleep quality than those who do not co-sleep. In respect of the mothers’ mental health, there did not seem to be a statistically significant difference in the two groups. Regarding the difficulties during the sleep process, children who sleep with their parents seem to have more difficulties compared to the others (*p* = 0.008). It was also shown that co-sleeping children seem to have more disturbed sleep compared to those who sleep alone (*p* = 0.018), and a general trend obtained of a significantly higher number of awakenings for co-sleeping children (*p* < 0.001). Finally, breastfeeding appeared to be more related to the children of the present sample sleeping with their parents (*p* < 0.001). Conclusions: This study showed that co-sleeping is associated with more difficulties in infant and maternal sleep, but no direct correlation with maternal mental health was found. In addition, it showed a positive correlation of co-sleeping with breastfeeding.

## 1. Introduction

One common parenting practice that has been of concern in the international literature is the use of the method of co-sleeping. Co-sleeping is defined as the parent and child sleeping in the same bed, but also sleeping in the same room even if the parent and infant sleep in different beds [1,2,3,4]. As the literature shows, most healthcare professionals working with infants do not prohibit co-sleeping if it is the parents’ wish, provided that all appropriate measures have been taken to ensure the safety of the infant [5]. Studies have shown that the practice of co-sleeping is largely determined by cultural and social norms and expectations [6,7]. A study on infant sleep in the first three years of life in 18 countries found that most infants (about 80%), mainly in Asian countries, share the same room with their parents, while about 50% of infants in Caucasian countries share the same room with their parents during the first months [6]. Despite the use of this method internationally, many conflicting opinions have been recorded regarding its implementation by parents during the first months of the child’s life.

Parents often choose co-sleeping for various reasons such as comforting a “difficult” infant, being next to an infant who is sick, to increase their bonding time with the infant, etc. In addition, co-sleeping supports the convenience of night-time breastfeeding and also reduces the pain of mothers due to getting out of bed, especially after a caesarean section [8]. Breastfeeding is, actually, one of the most important reasons why co-sleeping is chosen [8]. The importance of breastfeeding should not be underestimated in terms of its nutritional, immunological, and developmental benefits for both the mother and the infant. The American Academy of Pediatrics recommends that mothers should exclusively breastfeed their infants for the first six months of life and that breastfeeding should continue until at least the first year of life or as long as the mother and infant wish [9]. Regarding co-sleeping, mothers report easier breastfeeding, reduced fatigue as they do not have to get up, and a sense of security [10,11,12]. In addition, observational studies have shown increased breastfeeding rates, longer duration of breastfeeding, and better mother–infant interaction [13,14].

In contrast to the above, serious concerns have also been raised about the benefits or disadvantages of this practice. Co-sleeping has been found to be associated with an increased risk of sudden infant death syndrome and is therefore not recommended by many health professionals [15]. The American Pediatric Society in 2016 recommended that infants sleep in the same room as parents but on a separate sleeping surface to reduce the risk of sudden infant death syndrome [16].

In addition, co-sleeping has been associated with increased infant sleep problems. According to research, parents who practiced co-sleeping reported a delayed onset to bedtime, shorter duration of night sleep, more night awakenings, and were more likely to view their child’s sleep as a problem compared to parents who did not practice co-sleeping [6]. These findings are consistent with other studies suggesting that co-sleeping is associated with interrupted and less deep sleep for both parents and infants [17,18].

Infant/newborn sleep problems have often been associated from the parents’ perspective with increased anxiety, maternal depression within the family, reduced sense of competence, poor physical health, and reduced quality of life [19,20,21,22,23]. Maternal perinatal depression, defined as an episode of major depression occurring during pregnancy through the first 12 months postpartum, is a condition that affects up to 20% of women [24]. Perinatal depression has been associated with sleep problems in infants, shorter sleep duration, longer delay in bedtime, and more night awakenings [25]. In addition, depressed mothers, due to the sleep problems they experience, are likely to perceive that infant awakenings last longer [25]. Preventing infant sleep problems may be one way to reduce the rate of depression. This is very important, especially for mothers who are breastfeeding and often do not receive medication to treat depression [26].

Regarding co-sleeping, the problems that mothers experience in putting infants to sleep at the beginning of the night and putting them back to sleep during the night also disrupt their own sleep, resulting in them reporting severe fatigue [21], feeling less able to effectively care for the infant, and symptoms of maternal depression [19]. There is some research indicating a positive correlation between co-sleeping and depressive symptoms. In particular, a study conducted in Barbados found that mothers who co-sleep with their babies reported greater anxiety and hopelessness [27], while increased depressive symptoms were found in women living in Australia, Ireland, and the USA [20,28,29,30]. Although the association between co-parenting and the occurrence of depressive and anxiety symptoms has been studied to some extent, research is limited and there is no research indicating causality.

In the present study we aim to shed more light on the relationship between co-sleeping and maternal mental health with the ultimate goal of investigating causality. More specifically, this study aims to investigate whether the implementation of co-sleeping in infants aged 6–12 months (a) is associated with maternal complaints and mothers’ difficulties regarding their infant’s sleep, (b) is associated with maternal mental health, and more specifically, depressive and/or anxiety symptoms, (c) affects infant sleep characteristics and maternal sleep quality, and (d) is associated with breastfeeding.

## 2. Materials and Methods

### 2.1. Study Population

This study is a cross-sectional study conducted from July to November 2021. The participants of this cross-sectional study were the beneficiaries of a primary mental health care facility operating in Athens (Greece), namely the “Day Center for the Care of Women’s Mental Health (Perinatal Mental Disorders)”. The Day Center is the only specialized facility in the country for the prevention, early detection, and management/treatment of perinatal mental disorders. The Day Center was created in 2009 by “Fainareti”, a non-profit organization that aims to improve perinatal care in Greece through specialized midwifery and psychosocial interventions. The Center is supervised by the Mental Health Department of the Ministry of Health and all services are provided free of charge to the beneficiaries.

The inclusion criteria in this study were (a) primiparity, (b) age over 18 years, (c) having given birth 6–12 months prior, (d) having attended breastfeeding counseling sessions antenatally, (e) having received postnatal support by a midwife, (f) absence of drug use, and (g) absence of personal history of mental disorders. Of the total number of women who were using the Day Center’s services at the time of the survey, 151 new mothers participated.

Subsequently, all participants were divided into two different groups, the group of mothers who adopted the co-sleeping method from birth up to the time of the survey (6–12 months postpartum) and the group of those who did not adopt co-sleeping at that time. The first group included 98 mothers and the second group 53 mothers.

### 2.2. Procedure 

During the participant recruitment process, an invitation was sent by email to all new mothers who had given birth 6–12 months earlier and had attended the Day Center’s midwife-led prenatal educational program called “Preparation for Labor and Parenthood” (8–12 two-hour group sessions or 4–5 two-hour individual sessions). This program included information and counseling on the period of pregnancy, preparation for labor, education on breastfeeding, the needs, behavior, and adjustment of the baby, baby care-taking, emotional changes for a woman during the postpartum period, and the encouragement of maternal role and attachment development. The email the mothers received explained the purpose of the study, highlighted the confidentiality of the information and the anonymity of the questionnaires, and encouraged them to follow a link to the survey if they wished to participate.

This study was approved by the Research Ethics Committee of the Non-Profit Organization “Fainareti” (Ref.46/14 December 2020). All women had already signed and informed consent regarding the use of their data in future research during the registration process at the Day Center.

### 2.3. Measures

The data derived from (a) a questionnaire on mothers’ demographic and perinatal characteristics, infant feeding, and mothers’ sleep quality, (b) an instrument measuring infant sleep characteristic, and (c) two psychometric tools measuring depression and anxiety symptoms. The instrument/tools used are explained below.

#### 2.3.1. Infant Sleep Characteristics

##### Brief Infant Sleep Questionnaire—Revised Short Form (BISQ-R SF)

The BISQ-R SF is a revised standardized version of the Brief Infant Sleep Questionnaire designed by Sadeh [31]. The scale includes 20 specific items that assess infant and toddler (0–36 months) daytime and nighttime sleep patterns, as well as sleep-related behaviors and parent perception of the two weeks prior to completion date. It consists of three subscales: (a) Infant Sleep (IS) subscale, which assesses infant sleep patterns, (b) Parent Perception (PP) subscale, which assesses parental perceptions of their child’s sleep, and (c) Parent Behavior (PB) subscale, which assesses ecologically-based parental behaviors that have been found to impact infant sleep. Scores on each subscale and the total score are scaled from 0 to 100, with higher scores indicating better sleep quality, more positive perception of infant sleep, and parent behaviors that promote healthy and independent sleep [32]. This tool has not been validated for the Greek population. It was only translated for the needs of the study using English-back translation, which was then approved by the Pediatric Sleep Council. In this study, the average Cronbach’s alpha coefficient was 0.83.

#### 2.3.2. Maternal Mental Health

In our assessment of maternal mental health, we focused on depressive and anxiety symptoms, as these are the most prevalent disorders during the perinatal period [33]. To measure these symptoms, we employed the following self-report instruments:

(a) The Edinburgh Postnatal Depression Scale (EPDS)

The EPDS is a widely used screening tool designed by Cox et al. [34] that detects symptoms associated with prenatal and postnatal depression within the previous 7 days. It consists of 10 items, scored on a 4-point Likert scale (from 0 to 3). This screening tool has been translated and validated for the Greek population by two separate research groups [35,36] and has shown a very high overall internal consistency (Cronbach’s alpha for the total scale was equal to 0.80 in the validation study of Vivilaki et al. and 0.90 in the validation study of Leonardou et al.). The Greek version of the tool was used in the present study and the average Cronbach’s alpha coefficient was 0.83.

(b) State-Trait Anxiety Inventory (STAI)

The STAI is a self-administered scale that has been widely used to assess anxiety. It is a 40-item scale consisting of two 20-item subscales, one for state anxiety, i.e., the level of anxiety experienced at the time of assessment, and one for trait anxiety, i.e., the more general, longstanding tendency for experiencing anxiety. Each item is scored on a 4-point Likert scale ranging from 1 (almost never) to 4 (almost always), with greater scores indicating higher anxiety. The STAI has been translated into Greek and validated in the Greek population with adequate psychometric properties [37,38]. In this study, the average Cronbach’s alpha coefficient was 0.83.

### 2.4. Analysis

The data were analyzed using SPSS version 22.0. The description of quantitative variables was provided through mean values and the standard deviation, while absolute (*n*) and relative (*%*) frequencies were used for qualitative variables. Demographic factors included age, education, occupation status, and family financial management. Perinatal characteristics included child’s sex, prematurity, child’s age, type of feeding, duration of any and exclusive breastfeeding, and introduction of solid foods. The mother’s state characteristics included degree of fatigue, satisfaction with sleep quality, degree of feeling rested after sleep, anxiety, and depressive symptoms. The child’s bedtime characteristics included time of child’s bedtime preparation, number of child’s bedtime involvement of parents, bedtime difficulties, child’s sleeping problems, number of child’s awakenings, place of child’s sleep, duration of sleep with no awakening, morning awakening time, and number of daytime sleeps. In order to examine the relationship between the above characteristics and co-sleeping, multivariate analyses of variance (ΜANOVA) were applied. Chi-square (*χ*^2^) and binary logistic regression analysis were performed to examine the relationship between infant feeding and co-sleeping.

## 3. Results

Demographic and infant characteristics are presented in Table 1. The mean age of the sample was 35.62 ± 4.31 (*SD*) years, with almost an equal number of them having completed postgraduate (*N* = 73, 48.0%) and undergraduate studies (*N* = 69, 45.4%). More than half of the participants were private-sector employees (*N* = 73, 48.7%), while 12.5% (*N* = 19) were unemployed, and more than half of the sample reported easy family financial management (55.9%, *N* = 84). 

The number of boys in the current study was 67 (44.9%) and of girls 84 (55.1%), with seven of them (4.6%) having been born prematurely. The average age of the children was 8.18 ± 2.18 (*SD*) months. The percentage of children in the co-sleeping group that were being only and/or mainly breastfed was 72.4% (*N* = 71), while in the no co-sleeping group this applied to 39.6% (*N* = 21) of the children. 

Mother’s state and child’s bedtime characteristics are presented in Table 2. The majority of the participants, from both groups, reported a moderate and quite high degree of fatigue (*N* = 110, 72.3%). In addition, a quite high proportion of them reported moderate satisfaction with sleep quality (*N* = 58, 38.2%) and after-sleep degree of rest (a bit to moderate—*N* = 107, 69.8%). Time of child’s bedtime preparation was similar for both groups, namely at 08:15–9:00 p.m. for 44.1% (*N* = 67) of the sample, while only 4.6% (*N* = 7) began preparations after 10:00 p.m. Almost all mothers were involved in the child’s bedtime seven nights per week (*N* = 145, 95.4%). 

The difference between the two groups is noticeable in the number of child awakenings. More specifically, in the co-sleeping group the highest percentage of the children were waking up 2–3 times per night (38.8%), while in the no co-sleeping group almost half of them where not waking up at all, or they did only one time during the night (45.3%). Also, bedtime difficulties were mostly low on the 5-degree scale for the no co-sleeping group (*N* = 21, 39.6%), and moderate for the other group (*N* = 39, 39.8%). The highest percentage of sleeping duration with no awakening was more than 8 h for the no co-sleeping group (*N* = 23, 43.4%), whereas, in the co-sleeping group, for 33.7% (*N* = 33) the duration of continuous sleep was 2–3 h and for 30.6% (*N* = 30) 4–5 h. 

In the no co-sleeping group, 43.4% (*N* = 23) of the children were awakening between 6:00–7:00 a.m., while in the co-sleeping group, 27.6% (*N* = 27) were awakening between 7:15–8:00 a.m. and 36.7% (*N* = 36) after 8:30 a.m. Finally, approximately half of the children in the no co-sleeping group slept twice during the day (*N* = 28, 52.8%), whereas almost half of the children in the co-seeping group slept three times (*N* = 47, 48.0%). Table 2 also shows the EPDS and the STAI scores measured in the present study. These scores showed no association with co-sleeping with regard to this sample.

Table 3 shows the dependent variables of the current study that emerged from the applied multivariate analyses of variance as statistically significant in relation to co-sleeping.

At the univariate level, the *F* criterions showed statistically significant relationships between children’s co-sleeping and seven dependent variables measured in the current study. In terms of the child’s age, there was a statistically significant difference among those who sleep with their parents and those who do not (*p* < 0.001), where younger children were associated with sleeping with parents, with 10.7% proportion of the variance explained. Regarding bedtime difficulties, children who co-sleep appear to have more difficulties compared to those who do not sleep together with parents (*p* = 0.008). The proportion of the variance explained was 4.7%. Analysis involving children’s sleeping problems showed that those who co-sleep seem to have more problematic sleep, compared to those who sleep alone (*p* = 0.018), explaining 3.7% of the variance. Furthermore, the results revealed an overall tendency of a significantly greater number of child’s awakenings for the children who co-sleep (*p* < 0.001). The proportion of the variance explained was 8.5%. The biggest proportion of the variance explained (13.9%), with regard to the present sample, appeared for the duration of the child’s sleep with no awakening, where those who sleep alone seem to sleep continuously for longer than those who sleep with their parents (*p* < 0.001). Daytime sleeps were significantly more for those who co-sleep (*p* = 0.041). The proportion of variance explained was 2.8%. Finally, with 3.0% proportion of the variance explained, this was the dependent variable of mother’s satisfaction with sleep quality (*p* = 0.033), revealing mothers who sleep together with their children to have less quality sleep, in relation to those whose children do not co-sleep. 

An analysis was performed using the statistical criterion chi-square (*χ*^2^) for two independent samples, in order to examine the relationship between newborn feeding and co-sleeping (Table 4). A statistically significant relationship emerged between co-sleeping and infant feeding (*p* < 0.001), with only or mainly breastfeeding being more associated with the children of the current sample who sleep with their parents. This result was confirmed by a binary logistic regression analysis, which showed that those children who sleep alone have four times (*p* < 0.001) fewer possibilities to breastfeed [*B* = 1.34 (logistic coefficient), *Exp(B)* = 4.01 (exponentiated coefficient), *R*^2^ = 0.134 (assessment of interpretive power)]. It appeared that co-sleeping explained 13.4% of the variance of the newborn feeding variable.

## 4. Discussion

For many years now, co-sleeping has been a very controversial topic in terms of its physical and emotional risks and benefits for the newborn/infant and their mother [39]. The use of this practice has often been criticized for increasing the risk of sudden infant death syndrome (SIDS) [40,41], but on the other hand, the international literature has linked co-sleeping with a positive effect on breastfeeding [42]. It is known that this practice is implemented in many cultures of the world [43]. The present study aims to investigate whether the practice of co-sleeping at 6–12 months of age (a) is associated with maternal complaints and mothers’ difficulties regarding their infant’s sleep, (b) is associated with maternal mental health, (c) influences infant sleep characteristics and the quality of maternal sleep, and (d) is associated with breastfeeding.

In this study, mothers who use the practice of co-sleeping report that bedtime is a difficult situation that they often cannot manage, and that infant sleep is generally a greater problem within their family. The present study is consistent with other studies in which it is shown that the greater sleep problems are mostly reported by mothers who co-sleep with their babies [44,45]. Also, the present study indicated reduced sleep quality of infants who co-sleep with their mothers as they have more awakenings during the night. A similar study conducted in Israel showed that co-sleeping infants had poorer sleep, more awakenings, and fewer continuous sleeps at 3–6 months [4]. Similar findings have been highlighted by several previous studies [28,44,45,46,47,48,49]. On the other hand, fewer studies suggest that there is no link between co-sleeping and infant sleep [14,50,51,52] or even associate it with higher levels of sleep at specific stages of infant development [18,53].

It is important to note that the results of studies that associate co-sleeping with sleep difficulties are influenced by cultural factors. Most of the research concerns the Western population where awakenings and non-independent infant sleep is considered a problem, and it is regarded as “necessary” to train infants in the skills of self-soothing and the ability to sleep on their own. Blunden et al. argue that the fact that late night awakenings and non-independent sleep are considered a problem is a social construct [54].

Also, in relation to these findings we should consider the factor of breastfeeding as there are studies that link breastfeeding with interrupted sleep [55,56]. Therefore, as most of the co-sleeping mothers in this study are breastfeeding, we cannot be sure whether the cause of the infant’s sleep problems stems from the co-sleeping practice itself or because of the baby wanting to breastfeed more often. In addition, when the baby is right next to the mother, active sleep or any transition from deep sleep may be seen by her as an awakening. This could also account for the discrepancy between our findings and those of studies employing objective measures such as actigraphy or video recordings, which have found no significant difference in night awakenings between co-sleeping and solitary sleeping infants [50,51]. Similar differences in findings have been observed in other studies that utilized both subjective and objective measures, highlighting the discrepancies between subjective perception and objective assessment [1,4]. Another possible explanation for this is that co-sleeping infants may experience more frequent brief awakenings that mothers notice and report, but which are not detected by actigraphy. Additionally, Teti and Crosby [57] noted that mothers with elevated depressive symptoms and anxiety about their infants’ night waking tended to spend more time with their infants during these periods, even when the infants were not distressed. This heightened attention was associated with mothers reporting a higher frequency of infant night waking. Also, in future research, the general sleep profile of the mother before childbirth should be investigated. For example, a study of 153 families showed that women who had co-sleeping and reported having greater sleep problems had sleep-related difficulties even before pregnancy [4,56].

As highlighted by our results, co-sleeping is positively associated with breastfeeding. More specifically, the present study indicated that infants who were co-sleeping were breastfed longer and for longer periods of time exclusively than those who were not co-sleeping. This finding is consistent with the findings of previous studies [42,58,59,60,61]. Research that has positively correlated breastfeeding and co-sleeping includes studies that have made recordings of infant sleep and breastfeeding (with videos) during the night and studies based on mothers’ reported statements [14,42,50,62]. There have been studies in many countries around the world such as the United States of America, Sweden, Great Britain, New Zealand, Malaysia, Ireland, Australia, the Netherlands, and others [8,27,28,55,63,64,65,66,67,68,69,70,71,72,73]. The relevant research indicates that infants who co-sleep with their mothers breastfeed three times more than those who do not co-sleep [62,74].

However, it appears that this relationship between co-sleeping and breastfeeding also depends on the culture of each country [75]. A study conducted in a United Kingdom biethnic population showed that the interaction between breastfeeding and co-sleeping was greater for white British than Pakistani mothers [76]. Therefore, further research is undoubtedly needed to determine the relationship between co-sleeping and breastfeeding.

One of the aims of this study was to investigate the relationship between co-sleeping and the mental health of new mothers [77]. The results of the present study did not indicate a relationship between co-sleeping and the occurrence of depressive or anxiety symptoms in mothers. In contrast, in the literature, we see that co-sleeping is associated with depressive symptoms and stress in new mothers. Research conducted in Australia [20], Ireland [28], and the USA [29,30] shows that women who co-sleep with their babies report higher levels of hopelessness, anxiety, or sadness.

As is the case with breastfeeding, the international literature shows that culture is an important factor when it comes to the effect of co-sleeping on mental health [78,79,80]. Moreover, the direction of the effect is also unclear. A mother who suffers from anxiety or depression may choose to keep the baby close to her to monitor it more closely [30], while on the other hand a co-sleeping mother who sees this habit as problematic could experience feelings of anxiety [81]. More generally, the international literature does not focus so much on the effect of co-sleeping on mental health but on the effect that reduced infant sleep and more frequent awakenings may have on the development of depressive and anxiety symptoms in mothers. In previous research, decreased infant sleep at night and more frequent infant awakenings were associated with higher levels of stress, depressive symptoms, and poorer self-reported health in mothers, compared to mothers who did not practice co-sleeping [19,20,21,22,23]. In addition, poor-quality infant sleep has been shown to affect parental sleep, family functioning, and increase parental stress [82,83]. There are studies confirming that depressive symptoms are, actually, the result of sleep deprivation and not the cause of maternal sleep problems [75,82,84,85].

The bidirectional relationship between co-sleeping and maternal mental health is complex. On the one hand, co-sleeping could exacerbate maternal anxiety and depression due to disrupted sleep and concerns about the baby’s safety [29]. Frequent awakenings and the need to attend to the child can lead to maternal sleep deprivation, which, in turn, heightens stress and depressive symptoms [19,20,21,22,23]. On the other hand, mothers with pre-existing anxiety or depression might choose to co-sleep as a coping mechanism to feel closer to and better monitor their infants, potentially alleviating some of their distress but also creating a cycle of dependency and increased vigilance that can impair sleep quality [30]. Future research should further explore this bidirectional relationship, potentially through longitudinal or qualitative designs, and investigate how reduced sleep, co-sleeping, and the total number of awakenings affect both maternal and child mental health. Understanding the nuanced ways in which co-sleeping interacts with maternal mental health can inform better support strategies for new mothers, potentially leading to improved mental health outcomes and family well-being.

The literature, research, and guidelines related to co-sleeping are mainly provided by pediatricians. As suggested by previous studies [86,87] and supported by the findings of the present study, future research on co-sleeping should be more holistic and involve anthropologists, developmental psychologists, and other professionals so that generic guidelines around co-sleeping can be avoided and more specific information can be provided depending on the child’s stage of development, the needs of the family, and the social environment. Also, in future research, there should be greater anthropological analysis to further study the importance of the role of cultural factors. In countries such as Japan, for example, even in modern times, co-sleeping is much more commonly selected by new mothers than in the US [88]. This finding could be culturally justified as Japan’s culture promotes collectivity, solidarity, and interdependence while, in contrast, the US promotes autonomy and independence [88]. However, as demographic conditions change, the corresponding values and socialization practices change. For example, more and more cultures are living in “societies” and fewer in “communities”, the position of the sexes is changing, and the tendency for children to be independent is growing. All this shows the great anthropological scope of the issue of co-sleeping, which creates a prominent need for further research, and which will escape from the narrow frameworks that have already been applied.

One strength of the present study is its comprehensive approach to examining co-sleeping practices. By simultaneously exploring a variety of variables, the study provides a holistic view of co-sleeping beyond immediate outcomes such as sleep quality and difficulties. It extends its investigation to broader implications, including maternal mental health and breastfeeding practices. This comprehensive assessment could enhance our understanding of the intricate interplay between co-sleeping behaviors and health outcomes for both mothers and infants.

One potential weakness of the study is the unequal distribution of participants between the co-sleeping and no co-sleeping groups, with 98 mothers practicing co-sleeping compared to 53 who did not. A more balanced sample size between the groups could strengthen the study’s ability to generalize findings and ensure that both co-sleeping and no co-sleeping practices are adequately represented in the analysis. Future research could benefit from efforts to recruit a more evenly distributed sample, thereby enhancing the reliability and validity of comparisons between these distinct sleep practices. However, it is worth noting that despite the difference in group sizes, the characteristics of the study’s participants were relatively homogeneous in terms of their sociodemographic characteristics, residential locations, and life phase, which may mitigate some concerns about the unequal group sizes.

This study was conducted in a specific primary mental health care setting. Therefore, the study sample is not considered strictly representative of the Greek population, as the participants were mainly from the Attica region (with few exceptions). However, considering that the present study took place in an area where almost 50% of the Greek population resides, we could certainly say that its results are of value because they do not concern a small/numerically limited community of women with specific characteristics, but come from a large percentage of the Greek population, which showed ethnic homogeneity. Furthermore, this research is based solely on the women’s own statements regarding the current state of infant sleep, with no recording of their baby’s sleep patterns and without considering possible health problems that could affect the baby’s sleep. Finally, the concept of co-sleeping is not completely defined. Therefore, both during the literature review and when administering questionnaires to participants, co-sleeping may describe sleeping on the same bed or just in the same room on a separate surface.

## 5. Conclusions

This study showed that co-sleeping is associated with more infant sleep difficulties but there was no direct association with maternal mental health. In addition, the study showed a positive association between co-sleeping and breastfeeding. Further research is required to investigate the causality of the findings and the relationship between infant sleep and women’s mental health as co-sleeping may be a modifiable factor.

## Figures and Tables

**Table 1:** Demographic and infant characteristics.

	Co-Sleeping			
	No		Yes	
Demographic Characteristics	*N/M*	*%/SD*	*N/M*	*%/SD*
Mother’s age	35.73	3.81	35.53	4.58
22–30	5	9.4	12	12.2
31–35	19	35.8	34	34.7
36–40	22	41.5	39	39.8
41–48	5	9.4	13	13.3
Total/missing	51/2	96.2/3.8	98	100.0
Education				
High School	5	9.4	3	3.1
Bachelor’s Degree	22	41.5	47	48.0
Postgraduate Studies	25	47.2	48	49.0
Total/missing	52/1	98.1/1.9	98	100.0
Occupation				
Unemployed	4	7.5	15	15.3
Public-sector employee	8	15.1	18	18.4
Private-sector employee	29	54.7	44	44.9
Freelancer	12	22.6	21	21.4
Total	53	100.0	98	100.0
Family financial managing				
Always difficult	-	-	2	2.0
Sometimes difficult	11	20.8	23	23.5
Not that well	15	28.3	16	16.3
Easy	27	50.9	57	58.2
Total/missing	53	100.0	98	100.0
Perinatal Characteristics				
Child’s sex				
Boy	31	58.5	36	36.7
Girl	22	41.5	62	63.3
Total/missing	53	100.0	98	100.0
Prematurity				
No	49	92.5	95	96.9
Yes	4	7.5	3	3.1
Total/missing	53	100.0	98	100.0
Child’s age (months)	9.14	2.65	7.66	1.17
Newborn feeding				
Only breastfeeding/mainly breastfeeding	21	39.6	71	72.4
Only formula/mainly formula	32	60.4	27	27.6
Total	53	100.0	98	100.0
Any breastfeeding duration (months)	7.06	3.98	7.04	2.63
Exclusive breastfeeding duration (months)	3.85	2.64	4.57	2.21
Solid food introduction (month)	5.70	0.47	5.68	0.82

Note. *M*—mean; *SD*—standard deviation; *N*—frequencies; *%*—relative frequencies.

**Table 2:** Mother’s state and child’s bedtime characteristics.

	Co-Sleeping			
	No		Yes	
Mother’s State Characteristics	*N/M*	*N/M*	*N/M*	*N/M*
Degree of mother’s fatigue	3.26	1.06	3.28	0.87
Not at all	3	5.7	2	2.0
A bit	9	17.0	16	16.3
Moderate	18	34.0	38	38.8
Quite much	17	32.1	37	37.8
Very much	6	11.3	5	5.1
Total	53	100.0	98	100.0
Mother’s satisfaction with sleep quality	3.15	1.10	2.76	1.07
Not at all	5	9.4	8	8.2
A bit	7	13.2	36	36.7
Moderate	22	41.5	36	36.7
Quite much	13	24.5	8	8.2
Very much	6	11.3	10	10.2
Total	53	100.0	98	100.0
Mother’s after-sleep degree of rest	3.04	0.92	3.03	0.94
Not at all	3	5.7	1	1.0
A bit	9	17.0	30	30.6
Moderate	27	50.9	40	40.8
Quite much	11	20.8	19	19.4
Very much	3	5.7	8	8.2
Total	53	100.0	98	100.0
EPDS	7.38	4.36	6.72	4.73
STAI state	47.87	4.24	47.98	3.71
STAI trait	45.25	8.87	45.14	6.60
Child’s Bedtime Characteristics				
Time of child’s bedtime preparation	20.59	0.78	20.78	0.86
7:00–8:00 p.m.	17	32.1	23	23.5
8:15–9:00 p.m.	22	41.5	45	45.9
9:15–10:00 p.m.	12	22.6	25	25.5
>10.00 p.m.	2	3.8	5	5.1
Total	53	100.0	98	100.0
Number of child’s bedtime involvement of parents (nights)	6.66	1.30	6.90	0.77
0	1	1.9	1	1.9
2	1	1.9	-	-
3	1	1.9	-	-
4	-	-	1	1.9
5	1	1.9	-	-
7	49	92.5	96	98.0
Total/missing	53	100.0	98	100.0
Bedtime difficulties	2.26	1.06	2.76	1.07
Not at all	13	24.5	12	12.2
A bit	21	39.6	27	27.6
Moderate	14	26.4	39	39.8
Quite much	2	3.8	13	13.3
Very much	3	5.7	7	7.1
Total/missing	53	100.0	98	100.0
Child’s sleeping problems	1.08	1.22	1.63	1.43
Not at all	22	41.5	33	33.7
A bit	16	30.2	15	15.3
Moderate	8	15.1	15	15.3
Quite much	3	5.7	25	25.5
Very much	4	7.5	10	10.2
Total/missing	53	100.0	98	100.0
Number of child’s awakenings	2.00	1.61	3.29	2.23
0–1	24	45.3	19	19.4
2–3	20	37.7	38	38.8
4	6	11.3	18	18.4
>5	3	5.7	23	23.5
Total/missing	53	100.0	98	100.0
Place of child’s sleep				
In his crib	41	77.4	23	23.5
In his own bed	10	18.9	3	3.1
In parents’ bed	1	1.9	42	42.9
In a cradle connected to parents’ bed	-	-	28	28.6
In a stroller	1	1.9	-	-
In a basket	-	-	2	2.0
Total/missing	53	100.0	98	100.0
Duration of sleep with no awakening (hours)	7.11	2.95	4.99	2.29
2–3	5	9.4	33	33.7
4–5	16	30.2	30	30.6
6–7	9	17.0	19	19.4
≥8	23	43.4	16	16.3
Total/missing	53	100.0	98	100.0
Morning awakening time (hour)	7.50	1.00	8.00	1.06
6:00–7:00 a.m.	23	43.4	27	27.6
7:15–8:00 a.m.	17	32.1	35	35.7
≥8:30 a.m.	13	24.5	36	36.7
Total/missing	53	100.0	98	100.0
Number of daytime sleeps	2.40	0.82	2.67	0.82
1	5	9.4	7	7.1
2	28	52.8	31	31.6
3	14	26.4	47	48.0
4	6	11.3	13	13.3
Total/missing	53	100.0	98	100.0

**Table 3:** Multivariate analyses of variance of child’s co-sleeping.

	Child’s Co-Sleeping							
	No		Yes					
	*M*	*SD*	*M*	*SD*	*F*	*df*	*p*	*η* ^2^
Child’s age (months)	9.14	2.65	7.66	1.67	17.774	1	<0.001	0.107
Bedtime difficulties	2.26	1.06	2.76	1.07	7.335	1	0.008	0.047
Child’s sleeping problems	1.08	1.22	1.63	1.43	5.755	1	0.018	0.037
Number of child’s awakenings	2.00	1.61	3.29	2.22	13.798	1	<0.001	0.085
Sleep with no awakening (hours)	7.11	2.94	4.99	2.29	24.114	1	<0.001	0.139
Daytime sleeps	2.40	0.82	2.68	0.83	4.240	1	0.041	0.028
Mother’s satisfaction with sleep quality	3.15	1.10	2.76	1.07	4.644	1	0.033	0.030

Note. *M*—mean; *SD*—standard deviation; *F*—F criterion; *df*—degrees of freedom; *p*—statistical significance; *η*^2^—eta-squared index.

**Table 4:** Statistical criterion chi-square of child’s co-sleeping in relation to newborn feeding.

			Co-Sleeping			
			No	Yes	*χ*^2^(1)	*p*
Newborn Feeding	Only breastfeeding/ mainly breastfeeding	Count	21	71	15.57	<0.001
		Expected count	32.3	59.7		
	Only formula/ mainly formula	Count	32	27		
		Expected count	20.7	38.3		

## Data Availability

The appropriate data are written in the article.

## References

[1] Teti D.M., Shimizu M., Crosby B., Kim B.R. (2016). Sleep arrangements, parent-infant sleep during the first year, and family functioning. Dev. Psychol..

[2] Goldberg W.A., Keller M.A. (2007). Co-sleeping during infancy and early childhood: Key findings and future directions. Infant Child Dev..

[3] Li S., Jin X., Yan C., Wu S., Jiang F., Shen X. (2008). Bed- and room-sharing in Chinese school-aged children: Prevalence and association with sleep behaviors. Sleep Med..

[4] Volkovich E., Ben-Zion H., Karny D., Meiri G., Tikotzky L. (2015). Sleep patterns of co-sleeping and solitary sleeping infants and mothers: A longitudinal study. Sleep Med..

[5] Woods N.K. (2016). “Same Room, Safe Place”. J. Prim. Care Community Health.

[6] Mindell J.A., Sadeh A., Wiegand B., How T.H., Goh D.Y.T. (2010). Cross-cultural differences in infant and toddler sleep. Sleep Med..

[7] Mindell J.A., Telofski L.S., Wiegand B., Kurtz E.S. (2009). A nightly bedtime routine: Impact on sleep in young children and maternal mood. Sleep.

[8] Ball H.L. (2002). Reasons to bed-share: Why parents sleep with their infants. J. Reprod. Infant Psychol..

[9] Eidelman A.I., Schanler R.J., Johnston M., Landers S., Noble L., Szucs K., Viehmann L., Section on Breastfeeding (2012). Breastfeeding and the Use of Human Milk. Pediatrics.

[10] Tully K.P., Holditch-Davis D., Brandon D. (2015). The Relationship Between Planned and Reported Home Infant Sleep Locations Among Mothers of Late Preterm and Term Infants. Mater. Child Health J..

[11] Ball H.L., Hooker E., Kelly P.J. (1999). Where Will the Baby Sleep? Attitudes and Practices of New and Experienced Parents Regarding Cosleeping with Their Newborn Infants. Am. Anthropol..

[12] Hooker E., Ball H.L., Kelly P.J. (2001). Sleeping like a baby: Attitudes and experiences of bedsharing in Northeast England. Med. Anthropol..

[13] McKenna J.J., Mosko S.S. (1994). Sleep and arousal, synchrony and independence, among mothers and infants sleeping apart and together (same bed): An experiment in evolutionary medicine. Acta Paediatr..

[14] Baddock S.A. (2006). Differences in Infant and Parent Behaviors During Routine Bed Sharing Compared with Cot Sleeping in the Home Setting. Pediatrics.

[15] American Academy of Pediatrics Task Force on Sudden Infant Death Syndrome (2005). The Changing Concept of Sudden Infant Death Syndrome: Diagnostic Coding Shifts, Controversies Regarding the Sleeping Environment, and New Variables to Consider in Reducing Risk. Pediatrics.

[16] Knopf A. (2016). Babies on backs to sleep, but in same room as parents: AAP. Brown Univ. Child Adolesc. Behav. Lett..

[17] Thoman E.B. (2006). Co-sleeping, an ancient practice: Issues of the past and present, and possibilities for the future. Sleep Med. Rev..

[18] Mosko S., Richard C., McKenna J., Drummond S. (1996). Infant Sleep Architecture during Bedsharing and Possible Implications for SIDS. Sleep.

[19] Wake M., Morton-Allen E., Poulakis Z., Hiscock H., Gallagher S., Oberklaid F. (2006). Prevalence, stability, and outcomes of cry-fuss and sleep problems in the first 2 years of life: Prospective community-based study. Pediatrics.

[20] Hiscock H., Wake M. (2001). Infant sleep problems and postnatal depression: A community-based study. Pediatrics.

[21] Bayer J.K., Hiscock H., Hampton A., Wake M. (2007). Sleep problems in young infants and maternal mental and physical health. J. Paediatr. Child Health.

[22] Martin J., Hiscock H., Hardy P., Davey B., Wake M. (2007). Adverse associations of infant and child sleep problems and parent health: An Australian population study. Pediatrics.

[23] Smart J., Hiscock H. (2007). Early infant crying and sleeping problems: A pilot study of impact on parental well-being and parent-endorsed strategies for management. J. Paediatr. Child Health.

[24] Gelaye B., Rondon M.B., Araya R., Williams M.A. (2016). Epidemiology of maternal depression, risk factors, and child outcomes in low-income and middle-income countries. Lancet Psychiatry.

[25] Armitage R., Flynn H., Hoffmann R., Vazquez D., Lopez J., Marcus S. (2009). Early developmental changes in sleep in infants: The impact of maternal depression. Sleep.

[26] Gentile S. (2007). Use of Contemporary Antidepressants during Breastfeeding. Drug Saf..

[27] Galler J.R., Harrison R.H., Ramsey F. (2006). Bed-sharing, breastfeeding and maternal moods in Barbados. Infant Behav. Dev..

[28] Hughes A., Gallagher S., Hannigan A. (2015). A Cluster Analysis of Reported Sleeping Patterns of 9-Month Old Infants and the Association with Maternal Health: Results from a Population Based Cohort Study. Mater. Child Health J..

[29] Countermine M.S., Teti D.M. (2010). Sleep arrangements and maternal adaptation in infancy. Infant Ment. Health J..

[30] Teti D.M., Crosby B., McDaniel B.T., Shimizu M., Whitesell C.J.X. (2015). Marital and emotional adjustment in mothers and infant sleep arrangements during the first six months. Monogr. Soc. Res. Child Dev..

[31] Sadeh A. (2004). A Brief Screening Questionnaire for Infant Sleep Problems: Validation and Findings for an Internet Sample. Pediatrics.

[32] Mindell J.A., Gould R.A., Tikotzy L., Leichman E.S., Walters R.M. (2019). Norm-referenced scoring system for the Brief Infant Sleep Questionnaire—Revised (BISQ-R). Sleep Med..

[33] Kendig S., Keats J.P., Hoffman M.C., Kay L.B., Miller E.S., Simas T.A.M., Frieder A., Hackley B., Indman P., Raines C. (2017). Consensus bundle on maternal mental health: Perinatal depression and anxiety. J. Obstet. Gynecol. Neonatal Nurs..

[34] Cox J.L., Holden J.M., Sagovsky R. (1987). Detection of Postnatal Depression: Development of the 10-item Edinburgh Postnatal Depression Scale. Br. J. Psychiatry.

[35] Leonardou A.A., Zervas Y.M., Papageorgiou C.C., Marks M.N., Tsartsara E.C., Antsaklis A., Christodoulou G.N., Soldatos C.R. (2009). Validation of the Edinburgh Postnatal Depression Scale and prevalence of postnatal depression at two months postpartum in a sample of Greek mothers. J. Reprod. Infant Psychol..

[36] Vivilaki V.G., Dafermos V., Kogevinas M., Bitsios P., Lionis C. (2009). The Edinburgh Postnatal Depression Scale: Translation and validation for a Greek sample. BMC Public Health.

[37] Liakos A., Giannitsi S. (1984). The reliability and validity of the greek version of Spielberger’s State-Trait Anxiety Inventory. Enkefalos.

[38] Fountoulakis K., Papadopoulou M., Kleanthous S., Papadopoulou A., Bizeli V., Nimatoudis I., Iacovides A., Kaprinis G.S. (2006). Reliability and psychometric properties of the Greek translation of the State-Trait Anxiety Inventory form Y: Preliminary data. Ann. Gen. Psychiatry.

[39] Sadeh A., Tikotzky L., Scher A. (2010). Parenting and infant sleep. Sleep Med. Rev..

[40] Blair P.S., Fleming P.J., Smith I.J., Platt M.W., Young J., Nadin P., Berry P.J., Golding J. (1999). Babies sleeping with parents: Case-control study of factors influencing the risk of the sudden infant death syndrome Commentary: Cot death—The story so far. BMJ.

[41] Carpenter R., Irgens L., Blair P., England P., Fleming P., Huber J., Jorch G., Schreuder P. (2004). Sudden unexplained infant death in 20 regions in Europe: Case control study. Lancet.

[42] Baddock S.A., Purnell M.T., Blair P.S., Pease A.S., Elder D.E., Galland B.C. (2019). The influence of bed-sharing on infant physiology, breastfeeding and behaviour: A systematic review. Sleep Med. Rev..

[43] Nelson E.A.S., Taylor B.J., Jenik A., Vance J., Walmsley K., Pollard K., Freemantle M., Ewing D., Einspieler C., Engele H. (2001). International Child Care Practices Study: Infant sleeping environment. Early Hum. Dev..

[44] Mao A., Burnham M.M., Goodlin-Jones B.L., Gaylor E.E., Anders T.F. (2004). A Comparison of the Sleep? Wake Patterns of Cosleeping and Solitary-Sleeping Infants. Child Psychiatry Hum. Dev..

[45] Jenni O.G. (2005). A Longitudinal Study of Bed Sharing and Sleep Problems among Swiss Children in the First 10 Years of Life. Pediatrics.

[46] Kozyrskyj A.L., Kendall G.E., Zubrick S.R., Newnham J.P., Sly P.D. (2009). Frequent nocturnal awakening in early life is associated with nonatopic asthma in children. Eur. Respir. J..

[47] Coulombe J.A., Reid G.J. (2013). What Do Preschool-Aged Children Do When They Wake at Night: Toward an Understanding of Night-Waking Behaviors Among Community Children. Behav. Sleep Med..

[48] Huang X.N., Wang H.S., Chang J.J., Wang L.H., Liu X.C., Jiang J.X., An L. (2015). Feeding methods, sleep arrangement, and infant sleep patterns: A Chinese population-based study. World J. Pediatrics.

[49] Kelmanson I.A. (2010). Sleep disturbances in two-month-old infants sharing the bed with parent(s). Minerva Pediatr..

[50] Ball H.L., Ward-Platt M.P., Heslop E., Leech S.J. (2006). Brown KA. Randomised trial of infant sleep location on the postnatal ward. Arch. Dis. Child..

[51] Burnham M.M. (2007). The ontogeny of diurnal rhythmicity in bed-sharing and solitary-sleeping infants: A preliminary report. Infant Child Dev..

[52] Buckley P., Rigda R.S., Mundy L., McMillen I.C. (2002). Interaction between bed sharing and other sleep environments during the first six months of life. Early Hum. Dev..

[53] Hunsley M., Thoman E.B. (2001). The sleep of co-sleeping infants when they are not co-sleeping: Evidence that co-sleeping is stressful. Dev. Psychobiol..

[54] Blunden S., Thompson K.R., Dawson D. (2011). Behavioural sleep treatments and night time crying in infants: Challenging the status quo. Sleep Med. Rev..

[55] Galbally M., Lewis A.J., McEgan K., Scalzo K., Islam F.A. (2013). Breastfeeding and infant sleep patterns: An Australian population study. J. Paediatr. Child Health.

[56] Quillin I.M., Glenn L.L. (2004). Interaction Between Feeding Method and Co-Sleeping on Maternal-Newborn Sleep. J. Obstet. Gynecol. Neonatal Nurs..

[57] Teti D.M., Crosby B. (2012). Maternal depressive symptoms, dysfunctional cognitions, and infant night waking: The role of maternal nighttime behavior. Child Dev..

[58] Baddock S.A., Tipene-Leach D., Williams S.M., Tangiora A., Jones R., Iosua E., Macleod E.C., Taylor B.J. (2017). Wahakura Versus Bassinet for Safe Infant Sleep: A Randomized Trial. Pediatrics.

[59] Pollard K., Fleming P., Young J., Sawczenko A., Blair P. (1999). Night-time non-nutritive sucking in infants aged 1 to 5 months: Relationship with infant state, breastfeeding, and bed-sharing versus room-sharing. Early Hum. Dev..

[60] Beijers R., Riksen-Walraven J.M., de Weerth C. (2012). Cortisol regulation in 12-month-old human infants: Associations with the infants’ early history of breastfeeding and co-sleeping. Stress.

[61] Buswell S.D., Spatz D.L. (2007). Parent-Infant Co-sleeping and Its Relationship to Breastfeeding. J. Pediatr. Health Care.

[62] McKenna J.J., Mosko S.S., Richard C.A. (1997). Bedsharing Promotes Breastfeeding. Pediatrics.

[63] DeLeon C.W., Karraker K.H., Hildebrandt K., Karraker K.H. (2007). Intrinsic and extrinsic factors associated with night waking in 9-month-old infants. Infant Behav. Dev..

[64] McCoy R.C., Hunt C.E., Lesko S.M., Vezina R., Corwin M.J., Willinger M., Hoffman H.J., Mitchell A.A. (2004). Frequency of Bed Sharing and its Relationship to Breastfeeding. J. Dev. Behav. Pediatrics.

[65] Blair P., Ball H.L. (2004). The prevalence and characteristics associated with parent-infant bed-sharing in England. Arch. Dis. Child.

[66] Ball H.L. (2007). Bed-sharing practices of initially breastfed infants in the first 6 months of life. Infant Child Dev..

[67] Blair P.S., Heron J., Fleming P.J. (2010). Relationship Between Bed Sharing and Breastfeeding: Longitudinal, Population-Based Analysis. Pediatrics.

[68] Clements M.S., Mitchell E., Wright S.P., Esmail A., Jones D.R., Ford R.P. (1997). Influences on breastfeeding in southeast England. Acta Paediatr..

[69] Howel D., Ball H.L. (2013). Association between Length of Exclusive Breastfeeding and Subsequent Breastfeeding Continuation. J. Hum. Lact..

[70] Santos I.S., Mota D.M., Matijasevich A., Barros A.J.D., Barros F.C.F. (2009). Bed-sharing at 3 months and breast-feeding at 1 year in southern Brazil. J. Pediatr..

[71] Ford R.P., Mitchell E.A., Scragg R., Stewart A.W., Taylor B.J., Allen E.M. (1994). Factors adversely associated with breast feeding in New Zealand. J. Paediatr. Child Health.

[72] Weerheijm K.L., Uyttendaele-Speybrouck B.F.M., Euwe H.C., Groen H.J. (1997). Prolonged Demand Breast-Feeding and Nursing Caries. Caries Res..

[73] Tan K.L. (2011). Factors associated with exclusive breastfeeding among infants under six months of age in peninsular Malaysia. Int. Breastfeed J..

[74] Huang Y., Hauck F.R.F., Signore C., Yu A., Raju T.N.K., Huang T.T.-K., Fein S.B. (2013). Influence of bedsharing activity on breastfeeding duration among US mothers. JAMA Pediatr..

[75] Racial and Ethnic Differences in Breastfeeding Initiation and Duration, by State—National Immunization Survey, United States, 2004–2008. www.cdc.gov.

[76] Ball H.L., Moya E., Fairley L., Westman J., Oddie S., Wright J. (2012). Bed- and sofa-sharing practices in a UK biethnic population. Pediatrics.

[77] Meltzer L.J., Mindell J.A. (2007). Relationship between child sleep disturbances and maternal sleep, mood, and parenting stress: A pilot study. J. Fam. Psychol..

[78] Broussard D.L., Sappenfield W.M., Goodman D.A. (2011). The Black and White of Infant Back Sleeping and Infant Bed Sharing in Florida, 2004–2005. Matern. Child Health J..

[79] Luijk M.P.C.M., Mileva-Seitz V.R., Jansen P.W., van IJzendoorn M.H., Jaddoe V.W.V., Raat H., Hofman A., Verhulst F.C., Tiemeier H. (2013). Ethnic differences in prevalence and determinants of mother-child bed-sharing in early childhood. Sleep Med..

[80] Salm Ward T.C., Ngui E.M. (2014). Factors Associated with Bed-Sharing for African American and White Mothers in Wisconsin. Matern. Child Health J..

[81] Cortesi F., Giannotti F., Sebastiani T., Vagnoni C., Marioni P. (2008). Cosleeping versus solitary sleeping in children with bedtime problems: Child emotional problems and parental distress. Behav. Sleep Med..

[82] Byars K.C., Yeomans-Maldonado G., Noll J.G. (2011). Parental functioning and pediatric sleep disturbance: An examination of factors associated with parenting stress in children clinically referred for evaluation of insomnia. Sleep Med..

[83] Meijer A.M., van den Wittenboer G.L.H. (2007). Contribution of infants’ sleep and crying to marital relationship of first-time parent couples in the 1st year after childbirth. J. Fam. Psychol..

[84] Lam P., Hiscock H., Wake M. (2003). Outcomes of Infant Sleep Problems: A Longitudinal Study of Sleep, Behavior, and Maternal Well-Being. Pediatrics.

[85] Golik T., Avni H., Nehama H., Greenfeld M., Sivan Y., Tauman R. (2013). Maternal cognitions and depression in childhood behavioral insomnia and feeding disturbances. Sleep Med..

[86] Douglas P.S., Hill P.S. (2013). Behavioral Sleep Interventions in the First Six Months of Life Do not Improve Outcomes for Mothers or Infants. J. Dev. Behav. Pediatr..

[87] McKenna J.J., Ball H.L., Gettler L.T. (2007). Mother–infant cosleeping, breastfeeding and sudden infant death syndrome: What biological anthropology has discovered about normal infant sleep and pediatric sleep medicine. Am. J. Phys. Anthropol..

[88] Latz S., Wolf A.W., Lozoff B. (1999). Cosleeping in Context. Arch. Pediatr. Adolesc. Med..

